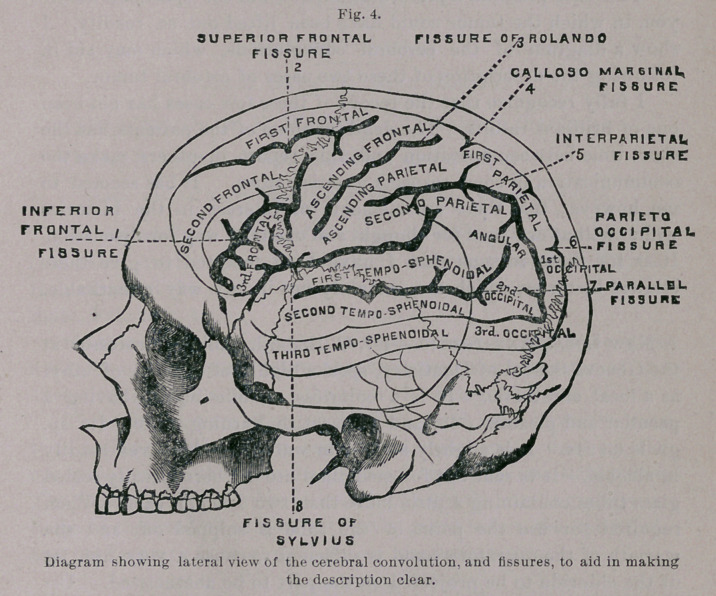# Clinical Contributions to Brain Surgery1Read at the stated meeting of the Philadelphia County Medical Society, November 25, 1891.

**Published:** 1892-02

**Authors:** John B. Roberts

**Affiliations:** Professor of Anatomy and Surgery in the Philadelphia Polyclinic; Professor of Surgery in the Woman’s Medical College of Pennsylvania


### CLINICAL CONTRIBUTIONS TO BRAIN SURGERY.[1]

By JOHN B. ROBERTS, M. D.,

Professor of Anatomy and Surgery in the Philadelphia Polyclinic; Professor of Surgery in the Woman's Medical College of Pennsylvania.

In 1885[2] I took strong ground in favor of more active surgical interference in injuries and diseases of the cranium and brain. At that time the views advocated by me were looked upon as being too radical, and were quite vigorously opposed by many prominent surgeons of this country. Since that date there has been developed an unprecedented activity in the operative treatment of cranial and intracranial lesions, which, even in my opinion, has been too extreme. It is, perhaps, not difficult to understand this unscientific and unreasonable adoption of what might be called a surgical fashion. It is to be regretted that the enthusiasm created by success impels some men to interfere surgically in nearly all cases that come into their hands, without a judicious study of each particular patient. That unrestrained mania for operating which has made abdominal surgery almost a by-word has, it seems to me, entered into the domain of cerebral surgery. It is just as much a part of scientific surgery to abstain from operating unnecessarily, as it is to combat vigorously the unreasonable conservatism of those who

1. Read at the stated meeting of the Philadelphia County Medical Society, November 25, 1891.

2. The Field and Limitation of the Operative Surgery of the Human Brain, *Annals of Surgery*, July and August, 1885.

will not see the force of anatomical, surgical, and statistical evidence. Fortunately for the patients, a healthy reaction is at last taking place, and surgeons are not now removing brain centers and tunnelling the brain in search of abscesses and tumors in quite as enthusiastic a manner as they were a couple of years ago. That such lesions should be promptly attacked surgically is unquestioned, but this should be done only after a thorough survey of the conditions and a judicial estimate of the gain that will possibly arise. The experimental character of many operations upon the brain in recent years, has been almost as patent as in vivisectal operations done with an avowed experimental purpose. Death on the operating table and unsuccessful operations have at length begun to stay the hands of these over-enthusiastic surgeons ; and there is now ground for hope that cerebral surgery will, ere long, become less reckless.

My personal opinions are very much what they were in 1885 ; indeed, the advances in diagnosis and the improvements in operative methods have made me even more sure of the correctness of the conclusions then advanced. I cannot, however, bring myself to approve of the reckless way in which human life is often threatened by operations which hold out scarcely a ray of hope to the helpless patient. The rapidity of healing in aseptic wounds and the tolerance of the brain under operative attack, do not justify hasty resort to intracranial surgery simply because the patient or his family are submissive under the persuasive eloquence of the would-be operator.

I desire tonight to report a few cases which have a practical bearing on some of the fundamental principles of cerebral surgery, and I hope they will serve as a means of bringing out the views of others in this interesting field.

CASE I. *Trephining for cortical epilepsy apparently the result of traumatism ; improvement, followed by death in five weeks.*—A child, twenty-nine months old, had sixteen months previously received a fall, and on the second day after the accident was seized with convulsions. Four months before he had been struck on the head by a falling clock, but no special symptoms followed this mishap. Since the second injury he had had spasmodic seizures occurring at frequent intervals during nearly every day. He dragged the left leg a little, did not seem bright, and was still unable to talk. There was a slight tendency to draw up the mouth on the left side, and also an inclination to turn the head and body to the left. When his attention was directed to bright objects he would, apparently, try to look at them, but his eyes usually turned to the left. His hearing seemed to be dull, but so far as could be determined the cutaneous sensibility was unimpaired. No changes were found by ophthalmoscopic examination.

Dr. Charles K. Mills, who referred the patient to me, placed the child under observation in order to detect, if possible, the exact character of the spasms. He was watched carefully in several seizures. Usually he squealed at the beginning of the paroxysm and his face had a vacant look. The spasm began with a lifting movement of the entire body, as if with the muscles of the trunk, much like a sudden effort to rise from a recumbent to a sitting position. About the same time, as nearly as could be judged, the eyes and head turned to the left. The eyes did not keep to the left, but oscillated with the jerking movements of the body; the head, however, continually turned to the left. The left leg and arm were spastic in slight flexion and were lifted up and projected outward and forward, The limbs on the right side were flaccid, but were projected forward and upward with the jerking movements apparently communicated from the trunk and the left limbs.

Another description of the attacks records that the child awakened suddenly from sleep with a toss of the body, as if badly frightened, with the head and eyes at once turning to the left. The left arm was extended forward and upward stiff and rigid, with the thumb and little finger pointing backward, the other fingers being slightly flexed. Both legs were also tossed upward in the air, the left more projected than the right. His body was lifted up and down during the attacks.

It was difficult to determine any signal symptom or serial order of movements. The spasm was both tonic and clonic, and certainly most marked in the limbs and face of the left side. The movements of the leg and arm were those of projection and protraction, and were rather movements from the shoulder and hip than from and in the distal portions of the limbs. The movements of the head, trunk, face, and limbs were often nearly coincident, but the conjugation of the head and eyes seemed certainly to be most commonly the initial movement.

The above description is taken from a former report of the case.[1]

Dr. Mills thought that the symptoms seemed to point to a lesion of the area for conjugate deviation of the head and eyes, and certain associated movements of the trunk, thigh, and arm. It was, therefore, determined to trephine over the posterior portions of the first and second frontal convolutions.

After encircling the head with a rubber bandage to prevent hemorrhage from the scalp, I made an opening with an inch and a half trephine placed one and a quarter inches in front of the fissure of Rolando and a little to the right of the median line. Behind and below the opening so made I cut out another button of bone with a one and a quarter inch

1. *Polyclinic*, April, 1889, p. 299.

trephine. The spurs of bone between the two holes were cut away with forceps. One point of the aura was abnormal in thickness and rather more adherent than normal. This condition did not seem to be caused by a Pacchionian body.

A flap of the dura was raised. The pia mater was very edematous, so that it could be pitted with the finger. A thin, yellowish-white membrane was found lying loosely upon the pia-archnoid and had probably separated from the dura when the flap of that membrane was raised. This abnormal membrane was removed. Small electrodes applied to the convolutions failed to induce contraction of the left arm. This electrical test was repeated, but failed to give results, though no antiseptic solution had come in contact with the brain tissue before the electrodes were used. Incisions in the pia allowed the serum which caused the edema to escape. When the convolutions were thus clearly exposed, there was no evidence of change in their structure or of any subjacent lesion. The dural flap was then sutured in position, and the portions of bone, which had been kept in antiseptic solution at a temperature of 105°, were replaced. Some catgut threads were laid beneath the buttons of bone and carried through the incision in the scalp to give drainage.

The child was under my observation for nineteen days, during which time there were only three epileptiform attacks and these were within two or three days after the operation. They were all slight and would scarcely have been recognized as pathological symptoms, if the previous severe attacks had not formed part of the clinical history. A large amount of cerebro-spinal fluid escaped for several days through the opening left by the catgut drain, which was removed a day or two after the operation, and also through a small hole in the line of incision which had not healed by first intention as had the rest of the wound.

Bromide of potassium, calomel, and small amounts of alcoholic stimulants were given to the child during the after-treatment.

When he was discharged from under my immediate care his general condition was good, temperature normal, and there had been no escape of cerebro-spinal fluid for three days. The two small openings in the scalp were covered with small crusts.

Two weeks later the child died, but the history of the intervening period is unknown. I heard only indirectly of his death. No post-mortem examination was made, but indefinite information has come to my knowledge, which leads me to believe that suppuration under the scalp occurred.

This case is one of a class in which there is a great temptation to operate in hope of finding some removable lesion of the cortical centers. The findings are usually negative; and the results only temporarily satisfactory, even when the patient entirely recovers from the lesions incident to the operation. Unless the localizing symptoms and signs are more definite than in this instance, I think that in similar cases I shall hereafter be almost inclined to avoid operative interference. This provisional conclusion has been reached by a consideration of cases in the treatment of which I have been concerned, or with whose results I am familiar.

CASE II. *Traumatic epilepsy resulting from unsuspected fracture; trephining with discovery of an irregular projection of bone on the interior of the cranium.*—A man, J. H., aged thirty-four years, while working as a puddler, about eight years ago, received an injury on the left side of the head by being caught between an iron lever of a furnace and a brick wall. He was treated by no physician, and only lost about two days from his work, although the injured region was poulticed by him, and was the seat of a discharge for four or five months. No portion of bone came from the wound, and there were no special symptoms.

Several years ago he had venereal sores upon the penis, but no suppurating inguinal glands or syphilitic developments. Chills and fever, several years ago, constituted the only illness from which he suffered.

An examination of his head, after shaving, revealed several insignificant scars, and just above the zygoma on the let side, a half inch in front of the auricle, a depressed cicatrix sufficiently deep to hold the tip of the little finger. This was the scar left by the injury received eight or nine years ago. The cicatrix involved the temporal muscle, as was seen by the dragging of the skin over the scar during mastication. There was no evidence of depression of the skull in any other part of the cranium, and this depression did not seem to involve the underlying bone. His intelligence was good, but the patient said that he did not remember as well as he could a few years ago, and that at times his eyesight was not good. He shows at times a little mental deterioration. An ophthalmoscopic examination of the eyes gave negative results.

The patient states that about two and a half years ago he had an epileptic fit after working in a hay-field on a hot day, and that since that time he has had marked seizures about every six weeks, with lesser attacks more frequently. He has but one epileptic fit at a time, from which he rapidly recovers, and is soon able to walk about. After such attacks he feels weak for some time. For several years he has had severe headache, not confined to any one portion of the head, and just before the epileptic seizure he feels a jerking sensation on the right side of the nose. He complains that his general health has deteriorated, but there is no apparent loss of flesh.

On the 26th of September of the present year (1891), I turned up a large flap of the scalp and found, after cutting through the temporal muscle, a depression in the skull one inch in length and three-eighths of an inch in width. This fracture was a surprise to me because of the history of the case and the situation of the injury over the thick belly of the temporal muscle. A three-quarter inch aeptic trephine was applied above and behind the depression. This cut through the bone with some difficulty, because the upper portion of the disc was much thicker than the lower part. Unfortunately my segment trephine had been forgotten, or this part of the operation could have been more expeditiously performed. Thinking I had cut entirely through the skull, I endeavored to pry out the disk, but removed simply the outer table of the button ; I found that between it and the internal surface there was a portion of fibrous tissue entangled. It was probably this portion of tissue entangled in the bony cicatrix as a result of the fracture at the time of the injury, that enabled me to lift out so readily the upper surface of the bony disk. The entangled tissue was doubtless pericranium. Removal of the interior table of the disc revealed below and in front of the opening a teat-like elevation projecting from the lower surface of the skull and pressing upon the dura. This elevation was about one-fourth of an inch higher than the general surface of the interior table, and was the apex of an irregular elevation due to consolidation of a number of comminuted fragments of the inner table. The irregular lines of fracture, with the fragments displaced in varying degrees, are shown on the button removed and the rest of the bone subsequently cut out with gnawing forceps.

The specimen shows this condition very satisfactorily, though somewhat mutilated by the gnawing forceps with which the adjacent bone was removed after the original button was taken out. The depth of the skull wound and the thickness of the temporal muscle made it rather difficult to operate neatly, and my desire to get rid of the portion of bone pressing upon the dura, without prolonging the operation or increasing its severity, caused me to sacrifice the specimen in the interest of the patient. The dura was not opened, threads of catgut were used for drainage, and a dry sublimate dressing was applied.

The following day the wound was found to be healing by first intention, and the drainage threads were removed. Bromide of potassium and chloral were given for two nights ; and then twenty grains of bromide of potassium three times a day were ordered as a continuous treatment.

On the third day after the operation the patient had a sensation of twitching at the side of the nose similar to that which formerly preceded the epileptic seizures ; but he had no fit. The wound healed by first intention, the temperature never rose above 99.6°, and on the eleventh day after the operation the patient was sent to his home in the center of the State. He felt exceedingly well after the operation and expressed his satisfaction at the improvement of his condition. I suggested that the bromide treatment be continued by his physician, Dr. J. P. McCleery, under the idea that removal of the surgical cause of epilepsy should be looked upon as only a part of the treatment. I believe that in all such cases internal treatment should be combined with surgical procedures, and that the epileptic habit should be controlled by a prolonged course of bromides after the mechanical cause has been removed.

Seven and a half weeks after operation his physician reported that

Fig. 1.

Outer surface of fractured cranium showing lambdoidal suture, point where trephine was applied, and small indentation looking like entrance of a vein made by the blow.

Fig. 2.

Inner surface of fractured cranium, showing cut made by trephine and large area of inner table driven inward under the small external indentation. The trephine has not cut entirely through the bone where the inner table is driven inward.

he had suffered no return of his epilepsy and was about to resume his work. As far as it goes, this statement is gratifying, but much more time must elapse before we can feel sure of a cure having been effected. The lesion is certainly one of those in which trephining ought to be eminently beneficial. Punctured fracture such as this should always be subjected to immediate trephining at the time of injury.

Above is given a representation of the external and internal appearance of the skull in a case trephined by me some years ago. There was a small scalp-wound through which I could, with my finger tip, feel what I thought was rough bone. I found by incision that the roughness was due to an unusually irregular lambdoidal suture with Wormian bones; and that the only bony lesion caused by the blow received from the pitcher, with which the patient was struck, was a small dent looking like the opening for the entrance of a vein. The character of the vulnerating force, however, induced me to trephine. The removal of the trephine button and the insertion of a probe between the dura and the cranium discovered nothing except a small fissure on the inner surface of the disc. Death occurred within a short time from alcoholic delirium; and the autopsy revealed a T-shaped fracture of the inner table with a shelf-like detachment of quite an area of bone. If this patient had lived he would probably have had secondary epilepsy, as occurred in the case just reported. The urgent necessity of primary trephining in such punctured fractures, even where no symptoms are present, is fully illustrated by these cases. The many deaths from cerebral abscess and other inflammatory processes, following the receipt of punctured fracture of the cranium, long ago justified the surgical conclusion that trephining in such injuries should not be delayed until the advent of symptoms of encephalic inflammation. The epilepsies resulting in cases which have escaped the immediate dangers of encephalitis, add another argument to the wisdom of immediate operation in punctured fractures.

CASE III. *Secondary trephining for traumatic epilepsy; death from aseptic cerebral inflammation.*—In June, 1891, I operated upon a man, J. T., aged twenty-eight, with the following history:

While working in a mine he had been struck upon the head with a huge mass of coal and rendered senseless. The attending physician. Dr. James D. Garvey, found a fracture of the skull, and upon the day of the injury removed a portion of the bone. According to the patient's statement he recognized no one for fourteen days, and was, therefore, probably unconscious during that time.

After consciousness returned his left arm was paralysed, but gradually regained power. Eight months afterward he had an epileptic seizure, and has had epileptic paroxysms at irregular intervals ever since. He is aware of the approach of a convulsion by nausea, dizziness and disorder of vision. Occasionally he has time, after the premonitory symptoms, to sit down before the fit occurs. He thinks that he ordinarily falls in the convulsion, but he does not bite his tongue at such times, though he froths at the mouth and grinds his teeth. The attacks have occurred as often as one or two in a day, but he has gone as long as four months without a paroxysm. The ophthalmoscopic examination reveals a normal fundus, clear media, and hyperopic refraction. He is unable to say in what part of the body the muscular spasm begins.

A large triangular depression is seen upon the right side of the head, the upper margin or base of which is one and three-quarter inches to the right of the median line and almost parallel to it. The apex of the triangle points downward and forward towards the ear. The anterior margin of the depression is near or a little behind the fissure of Rolando, and the center of the depression is over the superior parietal convolution, or in that vicinity. The deepest portion of the depression is that near the middle line of the skull, at which part its depth is fully a half inch ; the edge of the depression at this point is almost vertical. The inferior and posterior borders are less abrupt. The angle, which I have called the apex of the depressed triangle, is about two inches above the ear, and a little behind a vertical line drawn upward from the ear. The margins of the depressed area form an equilateral triangle, each side of which is about one and one-quarter inches in length. There are a number of other scars on the head, one or two of which radiate from this depression. There is distinct weakness of the grasp of the left hand, but no marked difference in size of the hand or the arm. The patient complains of the left hand feeling differently from the right. There is no muscular contracture and no apparent change in the electrical reaction or in mensuration.

On account of the epileptic attacks in this case I determined to operate and remove any apparent cause of irritation. If nothing abnormal was found, I intended to remove the cicatricial tissue in the bony gap and also the bony margin of the opening in the skull. Accordingly, I made an elliptical flap in the scalp, which disclosed a triangular depression in the skull corresponding with the indentation seen externally. This was filled in with fibrous tissue, which I dissected out of the bottom of the depression. The bone was so thick that the gnawing forceps could not cut away the edges ; hence, I used an aseptic trephine and removed a disc one inch in diameter from one corner. Subsequently I made four small holes along the edge of the depression with a half-inch trephine, and then was able to gnaw away the edges with gnawing forceps. The soft tissues were yellow, and pigmented in places with particles of carbon, evidently due to coal dust ground into the wound at the time of the accident.

Before the operation, pressure upon the scalp gave the sensation of a small cavity filled with air under the integument. It resembled the sensation experienced when a varicose vein is palpated. Removal of the skin over the gap in the cranium did not alter this tactile phenomenon. The yellow pigmented tissue, found as above mentioned, was not brain tissue ; and when cut through, disclosed what looked like the interior of an emptied cyst, because the inner surface of the tissue had a smooth, glistening surface. No fluid escaped or had escaped by puncture. After having dissected away a considerable portion of this material, and having removed the edges of bone along the entire circumference of the bony opening, I reached normal brain tissue. Hemorrhage from the cerebral wound and from the periosteum was profuse. It seemed impossible to stop that which came from the brain and its membranes, which were fused together in an almost indistinguishable mass at the bottom of the deep hole. The triangular opening in the skull measured about two inches along each margin. The pulse became very feeble, counting 165 a minute. Prolongation of etherization and operation seemed unwise.

After unsuccessful attempts to stop the bleeding by ordinary methods, I concluded to grasp all the bleeding points with hemostatic forceps which should be left in the wound. This was done, and five forceps were left in the wound with their handles protruding. Iodoform powder was dusted upon the surface of the exposed brain and strips of iodoform gauze packed into the cavity. A few sutures were applied after the flap had been replaced; the gauze strips and hemostatic forceps projected from one corner of the wound. A voluminous dressing of iodoform gauze and cotton was then applied and the patient put to bed. Seven and one-half hours after the operation the dressings were saturated with bloody serum, and, therefore, in order to avoid sepsis, I determined to reapply them and to remove the hemostatic forceps at the same time. This was done carefully, the gauze withdrawn, and the wound redressed with a dry antiseptic dressing. In drawing out the strips of gauze, a little oozing of blood occurred, but this hemorrhage I did not think of sufficient importance to prevent my closing the whole wound with sutures and without drainage.

The next morning the patient showed great restlessness, but was in a condition of hebetude. He, however, made his wishes known when he desired to urinate. Bromide and chloral were given to control the restlessness.

On the second day respiration varied from 25 to 40 in a minute, and the temperature was 101°. During the day the patient's condition was fairly good, though he was difficult to control on account of his restlessness and irritation. The urine was passed unconsciously. A turpentine enema was given; bromide and chloral were continued. On the third day after the operation it was necessary to give the patient one-sixth of a grain of morphine hypodermatically, and to strap him in bed because of his tossing from side to side. During the day he became hoarse, and I discovered at the base of the right lung harsh rales, probably bronchitic. The temperature was now 101.6°, while his respiration was between 35 and 40.

On the fourth day after the operation the note is made that he slept after a hypodermic of morphine, one-sixth of a grain, and is quieter. Respiration 40 to 45. His breathing, however, was embarrassed and harsh, somewhat of the Cheyne-Stokes's type. At 7 P. M. respiration was 50 ; temperature, 102°. The wound had been left undisturbed since the evening of the operation when the hemostatic forceps were removed. The rise in temperature and the patient's restlessness made me fear that there had been something amiss in my antiseptic precautions. I therefore determined to inspect the wound. Upon removing the dressing, I found the flap bulging and detected a feeling of fluctuation when my finger was put upon it. I expected to find pus under the flap, although the wound had healed by first intention. I tore open the union, but no evidence of pus existed ; a soft aseptic clot of blood, however, lay under the flap. I removed the clot and explored the cranial cavity through the operation wound with my finger in search for pus. The cerebral tissue was disintegrated and soft, but no purulent collection was found. I moved my finger in various directions in the pultaceous mass, and finally, when my little finger was buried its entire length, came upon a hard mass at the bottom. This, I presume, was one of the great ganglia. The tissue overlaying this dart was almost fluid. There was no odor of decomposition nor evidence of pus. At the time of this exploration the patient was moribund, and I felt fully justified in these radical measures. Unless I found pus he was sure to die.

The dressings were re-applied; hypodermic injections of strychnine were given. Respiration gradually failed and the patient died the next morning, which was the fifth day after the operation.

It seems hardly possible that the fatal symptoms were due to pressure from such a small amount of hemorrhage under the flap, since there was much space by reason of so much bone having been cut away ; and, moreover, the blood, if causing tension, would probably have readily escaped before the wound had united. I concluded, therefore, that death occurred from aseptic cerebral inflammation leading to disintegration and softening of the brain tissue. The pulmonary symptoms may have been secondary ; or he may have had a congestion, preliminary to an acute pneumonia, acting as a prominent feature in the fatal result. Rapid respiration was certainly an early symptom.

The case is to me exceedingly instructive, because the indications for operation were clear, and because death occurred notwith standing what seemed to be perfect aseptic conditions of the wound, during its entire course. It is a good illustration of the fact that modern surgery has not rendered serious operations entirely devoid of dangers. The diminution of the death-rate in operations has been great in recent years, but certainty of recovery is by no means as absolute as some reporters of operations would have us believe.

The next case is reported because of the youth of the patient.

CASE IV. *Trephining for depressed fracture of the skull in an infant seven months of age; recovery.*—A mother, while carrying her seven months' old child along a railroad track fainted or had an epileptic seizure, and fell, dropping the child. When she regained consciousness the baby was whining and fretting a little, but did not seem badly hurt. After the mother reached home and removed the child's wraps, she discovered a large indentation of the skull on the right side of the head, which she supposed was due to the child's head having struck against a railroad tie, or upon the iron track. The baby did not have any symptoms of brain implication.

When seen by me on the next morning, the infant was perfectly comfortable, had slept well all night, played as usual, and had a good appetite. The mother believed the depression to be less marked than when the accident occurred. Examination revealed an irregular depression in the parietal and occipital region on the left side of the head. The lower extremity of the vertical diameter of this depression was about two centimeters above and five centimeters back of the top of the ear. The depression extended upward six centimeters. The horizontal diameter—that is, that parallel to the sagittal suture—began at a point near the anterior portion of the posterior half of the parietal bone, and extended backward six centimeters, very nearly bisecting the vertical diameter. The depression at its deepest portion was fully a centimeter below the surface of the skull.

At this time the patient's temperature was normal; pulse, 120. During the night two grains of sodium bromide were given because of slight restlessness. The bowels were opened by a soap suppository.

On the second day after the accident I found the child feeling well and the depression less marked than on the previous day, when I made the first examination. I felt unwilling, however, to let the injury go without surgical treatment, and therefore determined to make at least an exploratory incision, because the injury had been so severe as to make a very deep depression. The possibility of secondary symptoms, such as epilepsy or impaired intellect, seemed to me to indicate this slight operative interference.

An Esmarch's bandage was carried around the head before the incision was made, to prevent bleeding. A horseshoe flap was then dissected up at the point of injury. The bone was markedly depressed, showing a condition similar to green-stick fracture. I thought I could cut through the cranium with a strong knife, but found it necessary to use a trephine. A small trephine opening was made through very thin bone at the anterior edge of the depression, and the portion pushed down upon the brain easily elevated with the end of a grooved director. A few bleeding arteries were twisted, and the edge of the scalp wound drawn together by catgut sutures. Boric acid powder and dry sublimate dressing was applied.

The patient re-acted from ether promptly and went quietly to sleep. Two-grain doses of sodium bromide were given at intervals until ten grains had been taken. The patient was restless through the night, but a few drops of paregoric quieted him. His bowels were kept open by injections of oil.

The temperature the day after the operation reached 101.8°, but soon all symptoms of fever disappeared, and on the seventh day the dressings were removed. The wound was found to have healed by first intention without suppuration.

At the end of the sixteenth day the patient was sent to his home in New Jersey entirely recovered.

In this case the accentuated character of the depression was the factor which led me to adopt operative procedures, although I know the tendency for depression of the skull in a healthy infant to correct itself spontaneously.

About eighteen months ago I saw a child who had received during birth a very marked indentation of the skull because the head had become locked on the promontory of the sacrum during delivery. The depression was situated on the left side of the head, and included portions of the frontal and parietal bones near the anterior fontanelle. It was about two and a half inches long and quite deep. The case was one of difficult labor, requiring forceps at the hands of Dr. Anna M. Fullerton, and the child, when born, was in the first degree of asphyxia, requiring the warm bath and artificial respiration. The child had frequent convulsions, beginning twenty-four hours after birth, evidently due to implication of the brain; yet I declined to operate, because I thought that the indentation was probably not associated with actual fracture of the soft bone. The convulsions ceased within twenty-four hours, and although the patient was under observation for several weeks, I never could convince myself that operative procedures were justifiable. The depression gradually lessened, and when the child was last examined by me seemed unimportant. The medicinal treatment of the child consisted of sodium bromide and potassium iodide. I have sometimes felt in regard to this case that the subsequent history might, perhaps, show that it would have been better to have interfered. I have not been able thus far to succeed in tracing the subsequent history of the little patient.

CASE V. *Specimen of cerebral tumor which could have been readily removed by surgical means.*—The brain herewith presented shows a tumor occupying the parietal region, and was obtained from a subject in the dissecting-room of the Woman's Medical College of Pennsylvania. The history of the case is, therefore, exceedingly indefinite, though through the courtesy of Dr. George S. Robinson I have been able to obtain the following notes:

The patient was a woman, aged thirty-five years, of intemperate habits, who had, so far as known, no injury of the head and was not discovered to be syphilitic. She was an inmate of a public institution and was sent to its infirmary about a week before her death, complaining of a pain in her head, which seemed to be somewhat relieved by pills of an anti-neuralgic character. The headaches continued, however, notwithstanding medication, and for about two days vomiting occurred. The patient then became comatose and paralysis of the right arm and leg supervened. The pupils were somewhat dilated and did not respond to light. Respiration was slow and the face flushed. No convulsions occurred, but there were slight twitching of the facial muscles. The patient was not noticed to be blind or deaf. Death took place on the sixth day after admission to the infirmary.

An examination of the specimen shows a flat, circular tumor in the right parietal region lying between the dura mater and the cerebral hemisphere. The convolutions are pushed downward by the growth, but are not infiltrated in the least degree. The dura has not been preserved, but it is quite evident that the growth was attached to the inner surface of the dura, since its upper surface is torn and it has no attachments to the convolutions, but can be lifted out of its bed without disturbing their integrity. The tumor is almost circular when inspected from above, being six centimeters in the antero-posterior diameter, and 6.5 centimeters in the transverse diameter. It is flat from above downward, varying from two to three centimeters in thickness. It occupies the right parietal region upon the superior aspect of the cerebrum. Its anterior margin lies in a line with the calloso-marginal fissure, and pushes forward the ascending parietal, or posterior central, convolution. The tumor extends backward to the parieto-occipital fissure, crowding downward and backward the first occipital convolution. It extends outward and downward to the posterior end of the parallel fissure, or the first temporo-sphenoidal fissure, pressing upon the angular gyrus. The first and second parietal convolutions are flattened and lie underneath the tumor in the concavity made by its growth producing pressure downward. On the inner aspect of the hemisphere the tumor presses the convolutions downward, being nearly two centimeters thick where it lay in contact with the falx. The anterior edge of the tumor is about one centimeter farther forward than the posterior edge of the corpus callosum. The gyrus fornicatus and the precuneus are pressed downward, but the cuneus does not appear to be pressed upon or displaced.

No surgeon can look upon this specimen without a feeling of regret that he could not have had an opportunity to attempt its removal. Its location immediately under the dura, its freedom from attachment to the cerebral convolutions, and its moderate size, would have made its removal easy. Its location behind the motor area is probably the reason that the patient's symptoms were not marked until just before the fatal termination of the disease. Her

Fig. 3.

Diagram showing relations of brain tumor. R, fissure of Rolando; I P, inter-parietal fissure; P O, parieto-occipital fissure: C M, calloso-marginal fissure. The tumor has been lifted out of its bed.

habits of life and surroundings were such that she would not be likely to call a physician's close attention to the early manifestations of cerebral disorder, if, indeed, these were apparent to the patient herself. A large opening made with trephine, gouge or saw, followed by a similar incision of the dura, would have enabled the operator to lift the tumor from its bed without hemorrhage or disturbance of the cerebral convolutions. The growth is probably a fibroma.

The occurrence of right-sided paralysis seems rather curious, but Dr. Robinson states that he is sure of the correctness of this note, for he remembers that she used her left hand during her final illness. There is no evidence of a second tumor on the left side.

Possibly the growth may have so pressed against the falx as to have impeded the current in the superior longitudinal sinus, and thus have given rise to pressure on the left cortical centers near the upper end of the fissure of Rolando. Unfortunately, I did not see the specimen until after the dura and falx had been removed.

CASE VI. *Probable basal cerebral tumor, in which operation was deemed inadvisable.*—In September, 1889, a man, aged thirty-four, was referred to me by Dr. H. C. Bloom, who had reached the conclusion that his patient was probably suffering with brain tumor. The history was somewhat difficult to obtain from the patient, who had evidently some impairment of mental faculties. In childhood he had had otorrhea in each side, and thought that his present ailments, of two or three years' duration, had succeeded a renewed discharge from the left ear. About a year before I saw him he had fallen insensible; but for a year and a half previously he had had attacks of severe pain in the head, to the left of the median line. Some failure of vision had been observed for eighteen months; occasionally he walks unsteadily, but there is no apparent loss of power in arms or legs. His family thought his mental traits had shown change for several years. He is now becoming fat, sleeps a good deal, and is somewhat "weak-minded" in his conversation and facial expression. There was no direct history of syphilis. Optic atrophy was found in both eyes, being more marked in the left, with which he could only see enough to count figures. The vision of the left eye was $\frac{4}{LX}$. Examination showed him to have lateral homonymous hemianopsia and Wernicke's pupillary re-action. The fields of vision indicated a left-sided lesion. No deviation of the eyes was determined, but he thinks he has at times had double vision. Both tympanic membranes were perforated. He had had no epileptic seizures, but, as above stated, had once fallen unconscious. The urine had a specific gravity of 1010 and contained neither albumin nor sugar. The grasp of the right hand was stronger than the left, accountable, perhaps, to his profession—that of a dentist. Thermometric examination for several days showed him to be free from fever.

No anesthesia nor paresis could be determined Dr. B. Alexander Randall's examination resulted in finding in the left ear an old cicatricial condition, with a mere trace of discharge. The original trouble had probably been present in childhood, and was now in abeyance, though occasional exacerbations had in all probability occurred The right ear was in a state of chronic suppuration of the attic and adjacent cavities, with some likelihood of the existence of diseased bone. No involvement of receptive or central auditory apparatus was discovered by the use of tuning forks. The patient's symptoms were thoroughly studied for me by Drs. Charles K. Mills, H. C. Wood, Edward Jackson, B. A. Randall, and A. W. MacCoy.

From Dr. William Osler, who had seen the man some months before, I learned that then he had had an intense optic neuritis, but at that time no hemianopsia. Dr. Osler suspected a slowly growing neoplasm; probably located in an interior location because of the early alteration in habits

Dr. Mills was inclined to think that the symptoms shown when the patient came under my care pointed to a lesion between the optic chiasm and the primary optic centers. This he considered might be a tumor or abscess of the inner part of the temporal lobe, encroaching on the optic tract back of the chiasm, or a similar lesion of the cerebellum advancing and invading the more anterior structures.

Dr. Wood believed the localizing symptoms pointed to a lesion encroaching upon the corpora quadrigemina or optic chiasm, which was most probably either a localized meningeal inflammation with much exudation, due to diseased bone at the base of the skull, or a tumor there situated. He thought it possible that an abscess might exist in the temporal or frontal lobe, but there was little evidence to indicate this being a probability.

Fig. 4.

Diagram showing lateral view of the cerebral convolution, and fissures, to aid in making the description clear.

This case was one that offered a good many points of surgical interest; but, after determining that the lesion was probably basal and on the left side, I declined to operate, because there was no evidence of the left ear being a probable cause of intra-cranial suppuration. If the symptoms had pointed at a right-sided lesion, the condition of the right ear would have influenced me strongly toward operative measures, looking to the evacuation of a temporal abscess. The association of chronic aural suppuration with cerebral abscess is so well known that I think I should have strongly inclined to exploratory trephining.

I accordingly declined to operate, and sent the patient home. I heard from him frequently, but he gradually lost vision and mental power. I had arranged for, and obtained permission for, an autopsy; but when he died the past summer no word was sent me. Previously to death he had violent pain in the head, a prolonged chill, several successive convulsions and coma with high temperature. These symptoms occurred suddenly and terminated fatally in four days. Before that time he thought his eyesight, which had been almost totally lost, was improving. The time he survived after my examination, nearly two years, leads me to believe that our abstinence from operation was correct, since the lesion was more probably a tumor than an abscess. If a tumor, its removal was certainly impossible.

This case presents a picture different from the specimen before you, in which the tumor could have been lifted out so readily. I show a diagram of the cerebral convolutions, which may aid in following the description of these two cases of cerebral tumor.

I fully recognize that the record of these few cases has not been one of brilliant results. The death of some of the patients, and the short time between operation and this report in others, make the communication in some respects unsatisfactory. It has seemed to me, however, that there are elements of interest in the histories which will afford food for thought and open the way for discussion. It is for these reasons that I have been tempted to give these clinical histories, which are certainly not in any way remarkable.

---

A New Local Anesthetic.—Dr. C. Redard, clinical professor at the Geneva School of Dentistry, speaks highly of chloride of ethyl as a local anesthetic. It is a colorless, mobile liquid, having a peculiar and pleasant odor and a sweetish burning taste. Its sp. gr. is 0.9214. It is slightly soluble in water, but dissolves readily in alcohol. It is sent out for medicinal use in hermetically sealed glass tubes containing a little more than two drachms each. When required for use the point of the tube is snipped off, and the warmth of the operator's hand is sufficient to cause a very fine jet of the chloride to be projected on the part to be anethetized. Up to the present its use has been confined to dentistry and as an external application in neuralgic affections, but there is little doubt that in a short time its value will be tested in general surgery. Its action is similar to that of methyl chloride.—*Scientific American, Oct.* 24, 1891.

---

The Topsy of an "Uncle Tom's Cabin" troupe died recently and bequeathed her body to the doctors. Autopsy!—*Texas Siftings.*

## Figures and Tables

**Fig. 1. f1:**
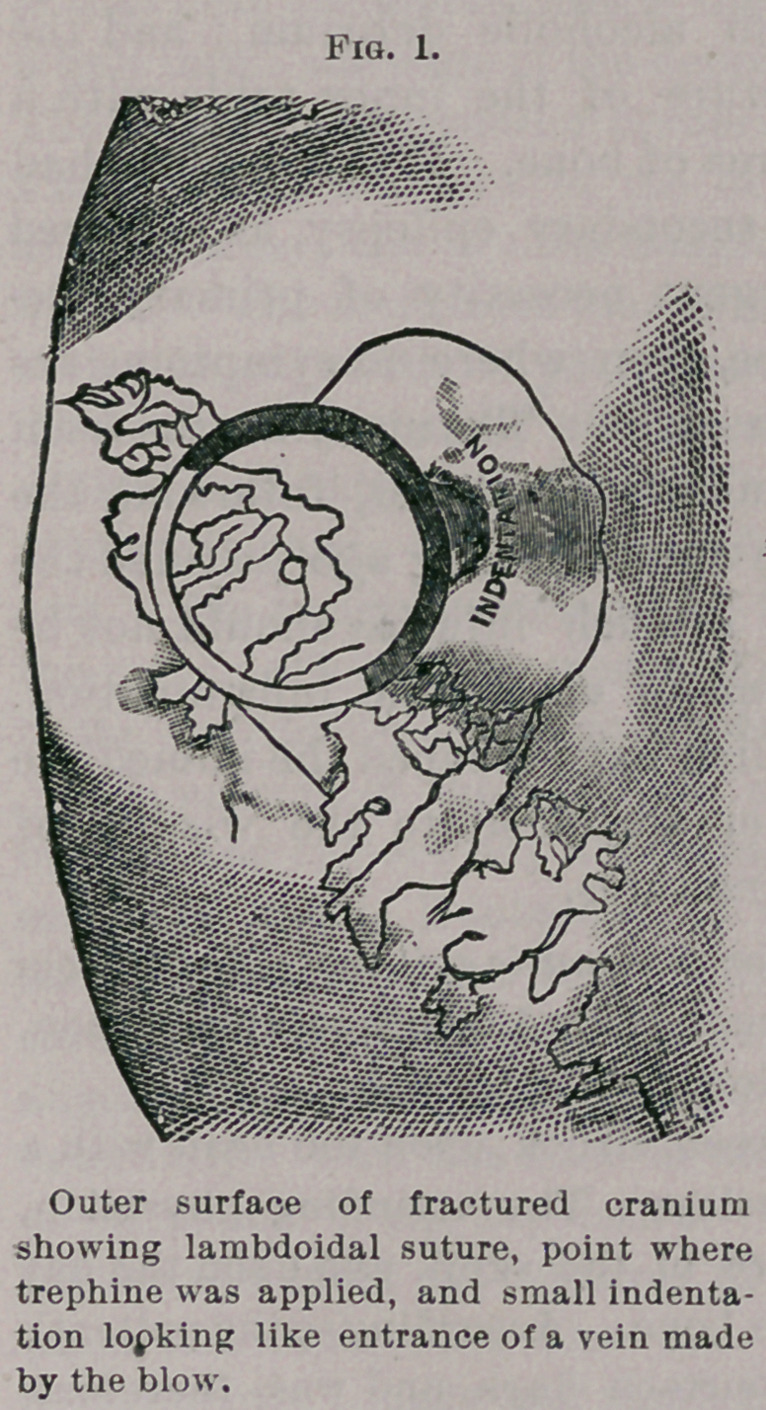


**Fig. 2. f2:**
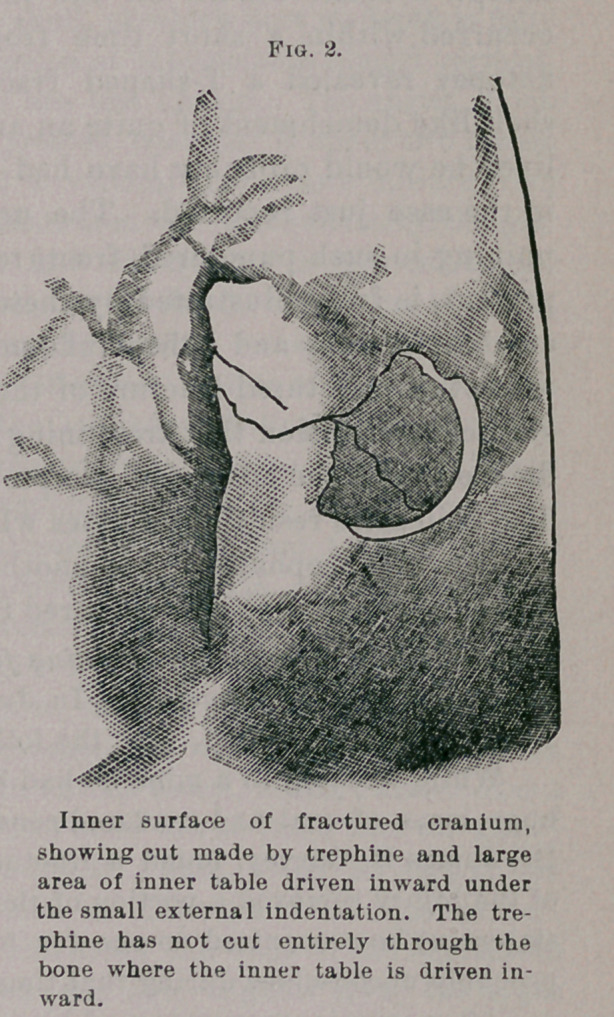


**Fig. 3. f3:**
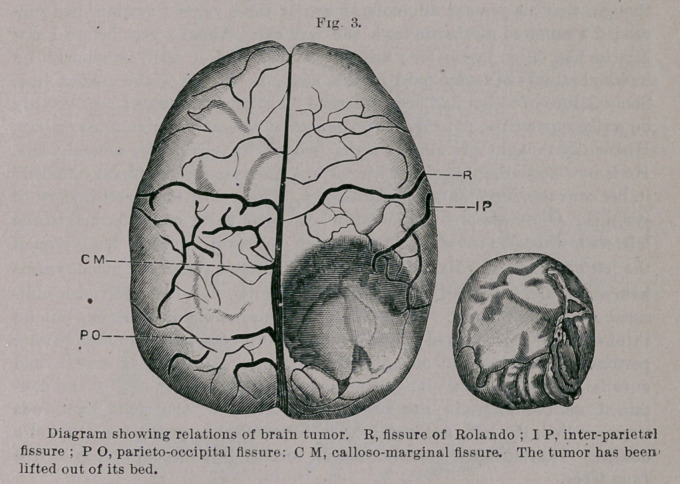


**Fig. 4. f4:**